# Prevalence and Risk Factors for Bladder and Bowel Dysfunction in Children With Type 1 Diabetes

**DOI:** 10.1155/pedi/5294835

**Published:** 2025-08-15

**Authors:** Kristen Favel, Maryellen S. Kelly, Shing Tat Theodore Lam, Jeffrey N. Bone, Kathryn E. Morgan, Heidi A. Stephany, Sruthi Thomas, Kourosh Afshar, Constadina Panagiotopoulos

**Affiliations:** ^1^Division of Pediatric Nephrology, Department of Pediatrics, University of California San Francisco, San Francisco, California, USA; ^2^Benioff Children's Hospital, San Francisco, California, USA; ^3^Division of Pediatric Urology, Department of Urology, Duke Health, Durham, North Carolina, USA; ^4^Division of Healthcare of Women and Children, School of Nursing, Duke University, Durham, North Caroline, USA; ^5^Department of Pediatrics, University of British Columbia, Vancouver, British Columbia, Canada; ^6^British Columbia Children's Hospital Research Institute, Vancouver, British Columbia, Canada; ^7^Division of Pediatric Urology, Department of Urology, University of Virginia, Charlottesville, Virginia, USA; ^8^Division of Pediatric Urology, Children's Hospital of Orange County, Orange, California, USA; ^9^Department of Urology, University of California Irvine, Orange, California, USA; ^10^Department of Urologic Sciences, University of British Columbia, Vancouver, British Columbia, Canada; ^11^Endocrinology and Diabetes Unit, British Columbia Children's Hospital, Vancouver, British Columbia, Canada

## Abstract

**Background:** Urologic complications, including urinary incontinence and urinary tract infections are commonly observed in the adult population with type 1 diabetes (T1D); however, there remains a paucity of data on the prevalence, associated risk factors and impact of bowel and bladder dysfunction (BBD) in the pediatric T1D population.

**Aim:** This study aims to examine the prevalence of BBD in children with T1D compared to healthy pediatric controls and to explore clinical factors associated with childhood BBD.

**Methods:** This cross-sectional, noninterventional, multicenter survey study involved children with TID and healthy controls aged 5–16 years across North America. Participants and their caregivers completed the Vancouver Symptom Score (VSS) to assess bowel and bladder symptoms. BBD was defined as a total VSS score of 11 or greater. Logistic regression was used to identify potential factors associated with BBD and bother with symptoms.

**Results:** In a group of 242 participants with T1D and 86 controls, 46% were male, and the median age was 11.0 years. The prevalence of BBD was found to be higher in participants with T1D at 21.5%, compared to 10.5% in controls. While irritative symptoms were most commonly reported in the T1D group with BBD, urinary incontinence caused the most bother. In the T1D group, poorer glycemic control was linked to a greater likelihood of BBD, while male sex and more severe symptomatology (such as urinary incontinence) were associated with greater bother related to these symptoms.

**Conclusion:** There is a high prevalence of BBD in children with T1D compared to healthy controls. These data highlight the need for early identification and intervention for BBD in T1D. Proactive measures, such as routine screening and comprehensive T1D management with strict attention to glycemic control, are crucial to address the significant burden of BBD and improve overall health outcomes for children with T1D and their families.

## 1. Introduction

In individuals with type 1 diabetes (T1D), chronic hyperglycemia may lead to a variety of long-term microvascular and macrovascular complications, including diabetic nephropathy, neuropathy, and retinopathy [1, 2]. Urologic complications, including urinary incontinence and urinary tract infections are also commonly observed in the adult population with diabetes and have been associated with significant impairment in health-related quality of life (HRQoL) [3–6]. Despite this, there remains a paucity of data on the prevalence, associated risk factors, and impact of urinary tract symptoms in the pediatric T1D population.

Bowel and bladder dysfunction (BBD) in children is a broad term that encompasses a range of symptoms, including lower urinary tract symptoms (also known as LUTS), encompassing urinary frequency, hesitancy, urgency, nocturia, and urinary incontinence, as well as fecal elimination issues, including constipation and fecal incontinence [7]. The etiology of BBD is often multifactorial, involving disruption of the neural control pathways and maladaptive voiding behaviors [8–10]. In the general pediatric population, the prevalence of BBD ranges from 5% to 20%, and is known to have significant impacts on psychosocial development and HRQoL for children and caregivers [11–14]; specifically, untreated BBD may lead to complications, such as urinary tract infections, renal scarring, and reduced kidney function [10]. Given the well-documented effects of diabetes on the peripheral and autonomic nervous systems [3, 15], and the high prevalence of LUTS in adults with T1D [6, 16], it stands to reason that children with T1D may be at heightened risk for developing BBD.

Given the importance of effective bladder and bowel function in growth, development, and overall well-being, a comprehensive understanding of the prevalence, risk factors, and consequences of these symptoms in children with T1D is critical to improving clinical care and quality of life in this vulnerable population. This study aims to examine the prevalence of BBD in children with T1D compared to healthy pediatric controls and to explore clinical factors associated with childhood BBD.

## 2. Materials and Methods

### 2.1. Study Design and Participants

This cross-sectional and noninterventional survey study was conducted at five pediatric centers (BC Children's Hospital, Duke University Children's Hospital, Children's Hospital of Orange County, University of Michigan CS Mott's Children Hospital, and University of Virginia Children's Hospital). Participants with T1D aged 5–16 years were recruited through the Diabetes and Endocrinology clinics. Control participants aged 5–16 years who were attending well-child visits were recruited from primary care clinics. Participants and families were either English or Spanish literate. Individuals with known current or active acute urinary tract infections, or anatomic or developmental anomalies that prevented the achievement of urinary and/or fecal continence, were excluded from the study. The Research Ethics Board for each site reviewed and approved the study protocol.

### 2.2. Study Instrument

All participants and their caregivers completed a validated questionnaire called the Vancouver Symptom Score (VSS) to assess bowel and bladder symptoms [17]. This 13-item Likert scale questionnaire evaluates four symptom subtypes as follows: urinary incontinence (UI; daytime and nocturnal urinary incontinence), irritative symptoms (IS; frequency, urgency, holding behaviors, dysuria, and nocturia), obstructive symptoms (OS; intermittency and hesitancy of urinary stream), and bowel dysfunction (BD; stool frequency and consistency, encopresis) (Table S1). A score of 11 or greater on the VSS indicates a diagnosis of BBD. For this study, two Likert scale questions were added to assess the degree of bother experienced by the child and caregiver; however, these do not contribute to the overall score. The VSS was administered independently to participants aged 9 years and older, while parents completed the questionnaire with participants younger than 9 years of age. The questionnaire was available in English and Spanish. Spanish translation of the entire questionnaire was completed and certified by Cryacom Language Services (Tucson, Arizona).

### 2.3. Outcome Definitions

The primary outcome variable, BBD, was defined as a total VSS score of 11 or greater [17]. Secondary outcomes included the prevalence of specific symptom subtypes (urinary incontinence, irritative symptoms, obstructive symptoms, and bowel dysfunction) in participants meeting the criteria for BBD, the prevalence of urinary incontinence in children with T1D compared to controls, and measures of caregiver/child bother associated with symptoms. Bother was defined as a child or parent reporting the symptom as “sometimes a bother” or “always a bother” (vs. “rarely a bother” or “never a bother”). Families were instructed to answer these bother questions separately to reduce influence.

### 2.4. Covariates

Data collected from clinic chart included age (at time of questionnaire), sex, body mass index for age and sex, and diabetes status (diagnosis of T1D). For children with T1D, the length of time in years from diagnosis of T1D (entered as a categorical variable), and hemoglobin A1c (at time of questionnaire) were also collected. Participants were categorized as normal weight, overweight, or obese according to the WHO growth reference standards for BMI z-scores (zBMI) [18, 19].

### 2.5. Statistical Analysis

Continuous variables were presented as median and interquartile range (IQR), while categorical variables were presented as number and percentage. Comparisons between means were done with the Student *t* test for normally distributed continuous variables, Wilcoxon test for nonnormally distributed continuous variables, and chi-square test for categorical variables. Logistic regression (using no BBD as the reference category) was used to identify potential factors associated with BBD and bother with symptoms. Variables were selected a priori based on clinical importance and included age and sex, and BMI, as well as A1c for participants with T1D. Participant age was chosen as a variable over the duration of diabetes due to the clinical importance of chronologic age in the context of bladder development and function. The effect estimates were reported as odds ratio (OR) and corresponding 95% CI. All the analyses were conducted in SPSS.

### 2.6. Missing Data

There were 63 participants missing at least one component of the VSS score. Of these, two were removed as they had all 13 components missing, 13 had scores ≥11 even with missing components and were marked as having BBD, 38 had scores <11 and even assuming the highest values for the missing components would result in a final score <11 and were therefore marked as nonBBD. Finally, the remaining 10 unclassified cases were excluded.

## 3. Results

In this study, 242 participants with T1D and 86 control participants were analyzed.  Table 1 highlights the characteristics of all study participants based on diabetes status. In participants with T1D, the duration of T1D was less than 5 years for 57% of participants, between 5 and 10 years for 31%, and 10 years or more for 12%.

### 3.1. Prevalence of BBD and VSS Scoring

The prevalence of BBD was found to be higher in participants with T1D at 21.5%, compared to 10.5% in control participants (*p*=0.03, Table 2). Participants with BBD had a higher median VSS of 13 (same in participants with T1D and controls), as compared to the VSS for participants without BBD (*p*  < 0.001, median VSS 6 in controls and 7 in participants with T1D).

### 3.2. Factors Associated With BBD in All Participants

Table 3 demonstrates factors associated with BBD for the entire study population. T1D was associated with a more than two-fold increase in the odds of BBD (OR, 2.34; 95% CI, 1.15–5.29). Older age was associated with lower odds of BBD, which decreased by 11% with each one-year increase in participant age (OR, 0.89; 95% CI, 0.82–0.98). The relationship with BMI showed increasing BBD with increasing weight, but confidence intervals included the null.

### 3.3. Factors Associated With BBD in Participants With T1D

**Figure 1:**
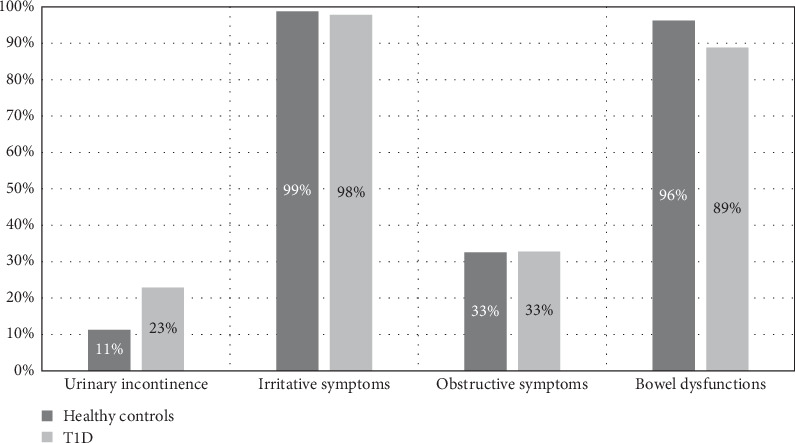
Breakdown of symptom prevalence in participants with BBD by diabetes status.

Among participants diagnosed with BBD, irritative symptoms were nearly universal in both the T1D and control groups (98% vs. 99%, respectively; Figure 1). Urinary incontinence more commonly reported among participants with T1D as compared to control participants (22.8% vs. 11.3%, respectively, *p*=0.03). There were no significant differences by sex based on symptom subtype of BBD.

Table 4 describes the factors associated with BBD in participants with T1D. Older age was similarly associated with lower odds of BBD in participants with T1D (OR, 0.89; 95% CI, 0.81–0.99). Elevated Hb_A1c_ was associated with a more than three-fold increase in the odds of BBD for A1c ≥9% (OR, 3.36; 95% CI, 1.32–8.74).

### 3.4. Factors Associated With Bother With BBD Symptoms in Participants With T1D

**Figure 2:**
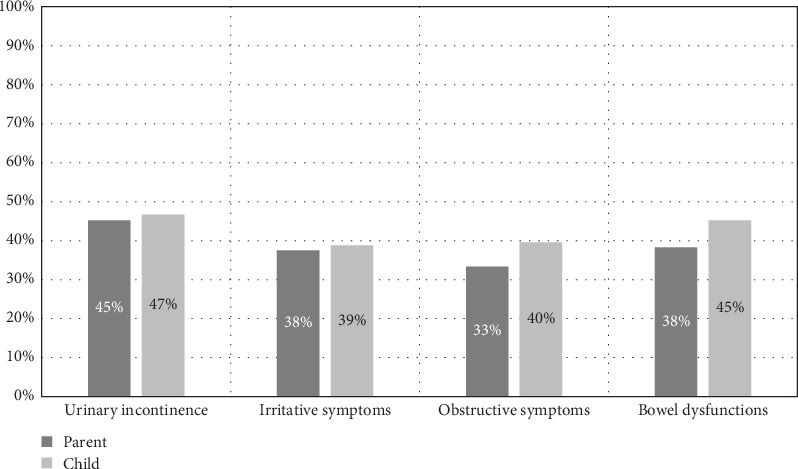
Symptom-related bother reported by participants with T1D and BBD and their parents.

More bother with symptoms was reported by participants with T1D vs. control participants (10.5% vs. 2.5%, *p*=0.04). Similarly, more bother with symptoms was reported by parents of participants with T1D vs. parents of control participants (11.7% vs. 5.4%, *p*=0.17). There was a similar frequency of bother by symptom subtype reported by parents and children (Figure 2). Bother with symptoms differed by sex, with higher odds of bother in males (OR 3.49; 95% CI, 1.45–9.29). Additionally, there was a 32% increase in the likelihood of bother with each one-point increase in VSS (OR 1.32; 95% CI, 1.20–1.47). Urinary incontinence was a less frequently reported symptom; however, it was the most bothersome symptom for children (OR 12.7; 95% CI, 5.12–34.9). There was no association between bother and age, A1c, and BMI *z*-score (Table S2).

## 4. Discussion

This study found several key insights. First, children with T1D had a higher prevalence of BBD compared to healthy controls. Second, while irritative symptoms were the most reported symptoms in the T1D group with BBD, urinary incontinence was the symptom that caused the most bother. Finally, in the T1D group, poorer glycemic control was linked to a greater likelihood of BBD, while male sex and more severe symptomatology (such as urinary incontinence) were associated with greater bother related to these symptoms.

This study builds on previous pilot research from Duke University Children's Hospital that found a significantly higher prevalence of LUTS in a combined pediatric type 1 and 2 diabetes population compared to healthy controls [20]. The higher prevalence of BBD observed in children with T1D compared to controls aligns with prior research in adult populations with T1D, which has found increased rates of urinary incontinence, urinary tract infections, and functional gastrointestinal disorders in this population [6, 16, 21, 22]. Much of the evidence on urologic complications in T1D has been derived from the Diabetes Control and Complications Trial and its observational follow-up study, the Epidemiology of Diabetes Interventions and Complications (EDIC) cohort. Notably, 15% of men and 16% of women experienced LUTS, and up to 30% of women experienced urinary incontinence after 17 years of follow-up as part of this EDIC cohort [23]. These adult studies have similarly found an association between elevated Hb_A1c_ and more frequent urinary symptoms and urologic complications, where it is postulated that hyperglycemia may contribute to autonomic dysfunction of the bladder, with symptoms further exacerbated by the direct effects of glucosuria and increased urine output [16, 21, 24].

In addition, this study highlights that BBD symptoms are a major inconvenience to children with T1D, with more bother reported by participants with T1D as compared to control participants. A growing body of pediatric evidence indicates that children with BBD and their caregivers experience poorer HRQoL, and children experience more frequent emotional and behavioral problems, particularly in cases of fecal and urinary incontinence [11–14, 25]. Similarly, adults with T1D and BBD have been found to have lower self-reported general health, physical functioning, and perception of quality of life compared to those without BBD [4]. Lower HRQoL due to BBD could contribute to suboptimal diabetes self-management, creating a cycle of worsening glycemic control, and BBD symptoms. Compounded by the impact of multiple comorbidities on HRQoL, like mental health conditions, the burden of BBD may further exacerbate difficulties faced by this population [26].

A key strength of this study was the use of a validated assessment tool (the VSS) to comprehensively evaluate the presence and severity of BBD symptoms. The study benefited from a large sample size and inclusion of a control group, allowing direct comparisons of BBD prevalence and associated factors. The cross-sectional design of our study precludes assessment of causality, and the lack of linkage to a HRQoL instrument limits a more direct evaluation of the effect of BBD on HRQoL, which warrants further exploration. Additionally, given the increased risk of comorbid mental health conditions in individuals with T1D, future studies should comprehensively evaluate the impact of mental health factors on the bother and distress associated with BBD symptoms [26–28]. Examining the relationship between mental health and the burden of BBD will be crucial to develop management strategies and improve the HRQoL for this patient population.

## 5. Conclusion

The findings from this study demonstrate that BBD is a distressing and common issue in pediatric T1D, which impacts both children and their caregivers. These data highlight the need for early identification and intervention for BBD in T1D, along with multidisciplinary clinical care with strict attention to glycemic control, education, and psychosocial support. Proactive measures, such as routine screening and comprehensive management strategies, are crucial to address the significant burden of BBD in this population and improve overall health outcomes for children with T1D and their families.

## Figures and Tables

**Table 1:** Participant demographics by diabetes status.

Characteristics	Type 1 diabetes (*n* = 242)	Healthy controls (*n* = 86)	All participants (*n* = 328)
Male sex	110 (45.5%)	43 (50.0%)	153 (46.6%)
Age, years	11.0 (9, 14)	11.0 (8.8, 14)	11.0 (9.0, 14.0)
Hb_A1c_^a^	7.8 (7.2, 8.7)	N/A	NA
z-BMI	0.69 (0.07, 1.43)	0.07 (−0.68, 1.00)	0.56 (−0.13, 1.34)
BMI classifications^b^			
Normal	145 (59.9%)	58 (67.4%)	203 (61.9%)
Overweight	45 (18.6%)	9 (10.5%)	54 (16.5%)
Obese	51 (21.1%)	9 (10.5%)	60 (18.3%)

*Note:* Median (IQR) reported for quantitative variables and absolute (%) for categorical variables.

^a^Hb_A1c_ not available for 1 participant with type 1 diabetes; Hb_A1c_ not collected for the control group.

^b^z-BMI calculated based on WHO classifications are not available for 11 participants (1 in T1D group and 10 in control group); overweight: 85th–<95th percentile; Obese ≥95th percentile [18, 19].

**Table 2:** Prevalence of BBD and VSS scoring.

Outcome	Type 1 diabetes (*n* = 242)	Healthy controls (*n* = 86)	All participants (*n* = 328)
BBD positive	52 (21.5%)	9 (10.5%)	61 (18.5%)
VSS	7 (5, 10)	6 (5, 8)	7 (5, 10)

*Note:* Median (IQR) reported for quantitative variables and absolute (%) for categorical variables.

**Table 3:** Factors associated with BBD.

Factors	OR (95% CI)
Diabetes status	
Control	1 (Reference)
T1D	2.34 (1.15, 5.29)
Male sex	1.33 (0.76, 2.33)
Age, years	0.89 (0.82, 0.98)
zBMI classification^a^	
Normal	1 (Reference)
Overweight	1.53 (0.70, 3.15)
Obese	1.78 (0.87, 3.53)

^a^z-BMI calculated based on WHO Classification and not available for 11 participants; Overweight: 85th–<95th percentile; Obese ≥95th percentile [18, 19].

**Table 4:** Factors associated with BBD in T1D.

Factors	Adjusted OR (95% CI)
Male sex	1.44 (0.74, 2.79)
Age, years	0.89 (0.81, 0.99)
Clinical Hb_A1c_ (%)	
Hb_A1c_ < 7.5	1 (Reference)
Hb_A1c_ 7.5–≤9	2.03 (0.95, 4.53)
Hb_A1c_ ≥9	3.36 (1.32, 8.74)
zBMI classification^a^	
Normal	1 (Reference)
Overweight	1.89 (0.81, 4.28)
Obese	1.52 (0.67, 3.36)

^a^zBMI calculated based on WHO Classifications; Overweight: 85th–<95th percentile; Obese ≥95th percentile [18, 19].

## Data Availability

The data that support the findings of this study are available from the corresponding author upon reasonable request.

## Supporting Information

Supporting Information Table S1. Vancouver Symptom Score Questionnaire. Table S2. Factors associated with bother of BBD symptoms in participants with T1D.
