# Supplementary material for: Prevalence and Risk Factors for Bladder and Bowel Dysfunction in Children With Type 1 Diabetes
Source: Pediatr Diabetes. 2025 Aug 15;2025:5294835. doi: 10.1155/pedi/5294835 (PMC12373478; doi:10.1155/pedi/5294835)
Supplement: Supporting Information — Table S1. Vancouver Symptom Score Questionnaire. Table S2. Factors associated with bother of BBD symptoms in participants with T1D. [file 5294835.f1.docx]

**Appendix**

| **Table S1.** Vancouver Symptom Score Questionnaire | | | | | |
| --- | --- | --- | --- | --- | --- |
| Questions | 0 | 1 | 2 | 3 | 4 |
| I pee in my underwear during the day. | Never | 1 day a week | 2-3 days a week | 4-5 days a week | Every day |
| When I pee in my underwear, they are. | I don't pee in my underwear | Almost dry | Damp | Wet | Soaked |
| In a normal day, I go to the bathroom to pee. | 1-2 times | 3-4 times | 5-6 times | 7-8 times | More than 8 times |
| I feel that I have to rush to the bathroom to pee. | Never | Less than half the time | Half of the time | More than half the time | Every day |
| I hold my pee by crossing my legs or sitting down. | Never | Less than half the time | Half of the time | More than half the time | Every day |
| It hurts when I pee. | Never | Less than half the time | Half of the time | More than half the time | Every day |
| I wet my bed at night. | Never | 3-4 nights per month | 1-2 nights per week | 4-5 nights per week | Every night |
| I wake up to pee at night. | Never | 3-4 nights per month | 1-2 nights per week | 4-5 nights per week | Every night |
| When I pee, it stops and starts. | Never | Less than half the time | Half of the time | More than half the time | Every day |
| I have to push or wait for my pee to start. | Never | Less than half the time | Half of the time | More than half the time | Every day |
| I have bowel movements. | More than once per day | Every day | Every other day | Every 3 days | More than every 3 days |
| My stool is hard. | Never | Less than half the time | Half of the time | More than half the time | Every day |
| I have bowel accidents in my underwear. | Never | 1-2 times per week | 3 times per week | 4-5 times a week | Every day |
| These symptoms are (Child response). | Never a bother | Rarely a bother | Sometimes a bother | Always a bother | --- |
| These symptoms are (Caregiver response). | Never a bother | Rarely a bother | Sometimes a bother | Always a bother | --- |
| A respondent with a score of 11 or greater is diagnostic of Bladder and Bowel Dysfunction (BBD) using the validated Vancouver Symptom Score (VSS)  survey, consisting of 13 4-item Likert scale questions. The two rate of bother questions do NOT contribute to the VSS but are used to evaluate whether  symptoms manifested as bother to the child and caregiver. | | | | | |

| Table S2. Factors Associated with Bother of BBD Symptoms in Participants with T1D | |
| --- | --- |
|  | Adjusted OR (95% CI)​ |
| Male Sex | 3.49 (1.45, 9.29)​ |
| Age, years | 0.95 (0.84, 1.07) |
| VSS Score | 1.32 (1.20, 1.47) |
| Urinary Incontinence | 12.7 (5.12, 34.9) |
| Clinical Hb_A1c_​ (%) |  |
| Hb_A1c_ < 7.5​  Hb_A1c_ 7.5 – ≤ 9  Hb_A1c_ ≥ 9 | 1 [Reference]  0.88 (0.32, 2.45)  2.34 (0.80, 6.91) |
| zBMI Classification^#^​ |  |
| Normal​  Overweight​  Obese​ | 1 [Reference]  2.05 (0.72, 5.48)  1.48 (0.49, 4.05) |
| ^#^zBMI calculated based on WHO Classifications; Overweight: 85th – < 95th percentile; Obese ≥ 95th percentile [18,19]. | |
